# Is cement-augmented sacroiliac screw fixation with partially threaded screws superior to that with fully threaded screws concerning compression and pull-out force in fragility fractures of the sacrum? – a biomechanical analysis

**DOI:** 10.1186/s12891-021-04933-y

**Published:** 2021-12-10

**Authors:** Juliana Hack, Maiwand Safi, Martin Bäumlein, Julia Lenz, Christopher Bliemel, Steffen Ruchholtz, Ludwig Oberkircher

**Affiliations:** Center for Orthopaedics and Trauma Surgery, University Hospital Giessen and Marburg GmbH, Baldingerstrasse, 35043 Marburg, Germany

**Keywords:** Cement augmentation, Fragility fracture of the pelvis, Fully threaded screw, Osteoporosis, Partially threaded screw

## Abstract

**Background:**

Providing a stable osteosynthesis in fragility fractures of the pelvis can be challenging. Cement augmentation increases screw fixation in osteoporotic bone. Generating interfragmentary compression by using a lag screw also improves the stability. However, it is not known if interfragmentary compression can be achieved in osteoporotic sacral bone by cement augmentation of lag screws.

The purpose of this study was to compare cement-augmented sacroiliac screw osteosynthesis using partially versus fully threaded screws in osteoporotic hemipelvises concerning compression of fracture gap and pull-out force.

**Methods:**

Nine fresh-frozen human cadaveric pelvises with osteoporosis were used. In all specimens, one side was treated with an augmented fully threaded screw (group A), and the other side with an augmented partially threaded screw (group B) after generating a vertical osteotomy on both sides of each sacrum. Afterwards, first a compression test with fracture gap measurement after tightening of the screws was performed, followed by an axial pull-out test measuring the maximum pull-out force of the screws.

**Results:**

The fracture gap was significantly wider in group A (mean: 1.90 mm; SD: 1.64) than in group B (mean: 0.91 mm; SD: 1.03; *p* = 0.028).

Pull-out force was higher in group A (mean: 1696 N; SD: 1452) than in group B (mean: 1616 N; SD: 824), but this difference was not statistically significant (*p* = 0.767).

**Conclusions:**

Cement augmentation of partially threaded screws in sacroiliac screw fixation allows narrowing of the fracture gap even in osteoporotic bone, while resistance against pull-out force is not significantly lower in partially threaded screws compared to fully threaded screws.

## Background

In times of an ageing society, osteoporosis and osteoporotic fractures are becoming more and more common [1, 2]. Due to the reduced bone quality and quantity in osteoporotic bone, providing a stable osteosynthesis is difficult. In particular, because of the weak screw anchorage in osteoporotic bone, lag screws cannot be used to generate interfragmentary compression.

A well-established technique for increasing stability of the osteosynthesis in osteoporotic bone is cement augmentation. The positive effects of cement augmentation concerning stiffness and stability of the osteosyntheses have been demonstrated in several studies [3–8].

However, hardly anything is known regarding the question of whether interfragmentary compression can be achieved by cement augmentation of lag screws in osteoporotic bone. In the current literature, only one study concerning this topic can be found: that of Wähnert et al., who performed a biomechanical study with cement-augmented lag screws in osteoporotic surrogate bones and demonstrated that cement augmentation can significantly improve interfragmentary compression [9].

In the treatment of osteoporotic fragility fractures of the sacrum, fully threaded screws are commonly used, mainly to improve the hold of the screw due to the longer thread [3, 6, 8, 10]. However, there are several potential advantages of using partially threaded lag screws in sacroiliac screw osteosynthesis, given the fact that interfragmentary compression can be generated. Inter alia, interfragmentary compression stimulates fracture healing, reduces pain due to less micromotion in the fracture gap, and increases the stability of the osteosynthesis [11–13].

However, to date no biomechanical studies exist concerning the question of whether cement-augmented lag screws can produce interfragmentary compression in osteoporotic sacral fractures, nor do studies exist comparing cement-augmented fully and partially threaded sacroiliac screw osteosynthesis.

Therefore, the purpose of this biomechanical study was to compare cement-augmented sacroiliac screw osteosynthesis using partially versus fully threaded screws in osteoporotic hemipelvises concerning compression of fracture gap and pull-out force. It was expected that cement-augmented partially threaded screws would increase interfragmentary compression of the fracture gap compared to fully threaded screws and thus can be used as lag screws. Furthermore, it was expected that the pull-out force of augmented partially threaded screws would be comparable to augmented fully threaded screws despite the shorter thread.

## Methods

### Specimen description

Nine fresh-frozen human cadaveric pelvis specimens (sacrum and posterior part of the ilium) were used.

The mean age of the donors was 68 years (SD: 6.98; min: 57; max: 76). All donors were male. Bone density was evaluated by dual-energy X-ray absorptiometry (DXA) and showed a mean T-score of − 3.75 (SD: 1.24; min: − 6.0; max: − 2.3), measured on the sacrum before preparation of the specimens.

Computed tomography (CT) scans of all pelvises showed no fractures or osteolytic lesions.

### Specimen preparation, fracture model and operative treatment

Fracture model and surgical treatment were based on the test setup of previous biomechanical studies of our research group [14, 15].

The pelvises were stored at − 20 °C. Before testing, they were prepared by removing all soft tissue except anterior and posterior sacroiliac ligaments, which were left intact. The specimens were thawed at room temperature before testing [15].

**Fig. 1:**
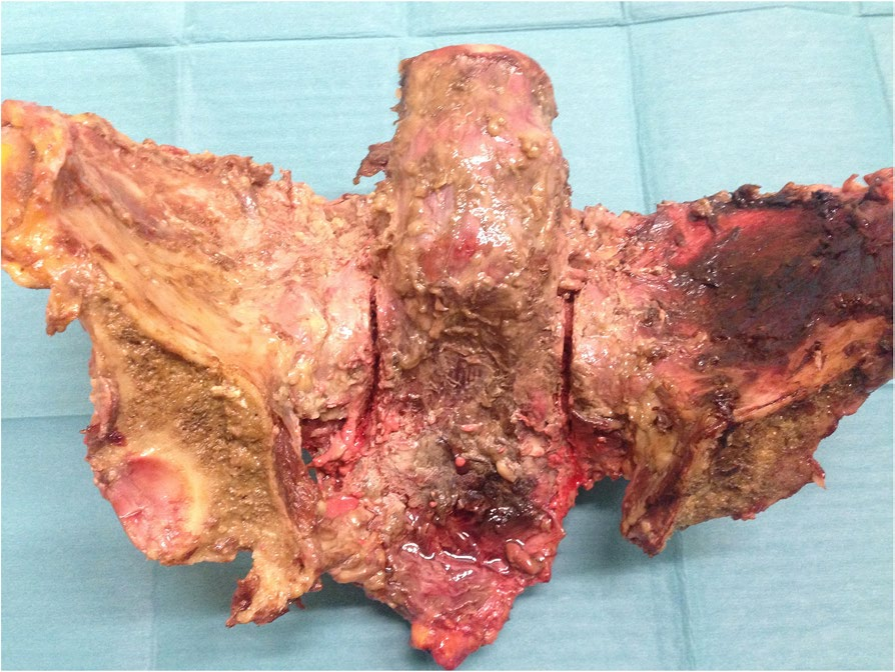
Vertical osteotomies on both sides of the sacrum, simulating the fractures

On both sides of the sacrum, a vertical osteotomy was generated in Denis zone I [16], using an oscillating saw (see Fig. 1). Each osteotomy was 4 mm wide (measured at the ventral edge of the first sacral vertebra using an electronic measuring gauge) and involved the complete craniocaudal length of the sacrum; the posterior cortex and the posterior sacroiliac ligaments were left intact [14, 15]. In this way, an FFP type-IIB fracture according to the classification by Rommens, namely a sacral crush injury with anterior disruption, was imitated (61-B3 fracture according to the OTA classification) [14, 15, 17, 18].

In all specimens, both sides of the sacrum were stabilized; specifically, one side was treated with a cement-augmented fully threaded screw (group A), and the other side with a cement-augmented partially threaded screw (group B), to directly compare both operative techniques in bone of the same quality. Consequently, each group consisted of nine specimens. In five of the specimens, the fully threaded screw was placed on the right and the partially threaded screw was placed on the left side of the sacrum; in the other four specimens, it was vice versa.

**Fig. 2:**
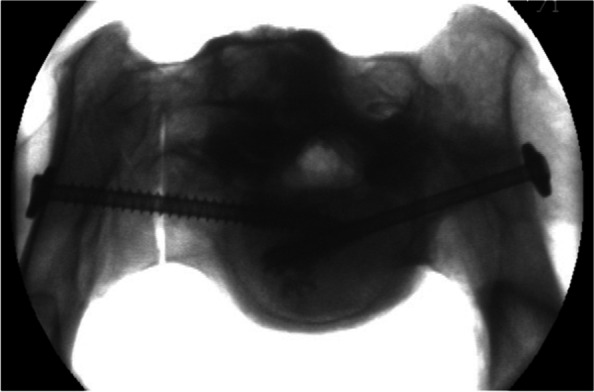
Fluoroscopic view of cement-augmented sacroiliac screw osteosynthesis with fully threaded screw on the right side and partially threaded screw on the left side

Operative treatment was performed as follows:

All operations were performed by the same surgeon (JH). In both groups, 7.5 mm cannulated, self-cutting lag screws (length 90 mm) made of titanium (aap Implantate AG, Berlin, Germany) were used [15]. In group A, fully threaded screws were used; in group B, partially threaded screws were used (32 mm thread length). Placement of the screws and cement application were monitored under fluoroscopy in three planes (a.p., lateral and craniocaudal; see Fig. 2).

First, a K-wire was positioned in the first sacral vertebral body under fluoroscopic guidance and overdrilled. A working cannula was placed for cement application (Vexim, Balma, France). Subsequently, the K-wire was removed, and 0.9 mL of bone cement (Cohesion® Bone Cement, Vexim, Balma, France) was injected at the end of the drill hole using a cement filler (Vexim, Balma, France). After that, the K-wire was reinserted, the cannula was removed and the cannulated screw plus washer was placed in S1 and tightened loosely [15]. Then, the K-wire was removed. After 5 min, the screws were tightened with a torque of 3 Nm, using an electronic screwdriver.

### Compression test

Fifteen minutes after tightening of the screws, the fracture gap was measured ventrally at the level of the screw on both sides of each sacrum, using an electronic measuring gauge.

### Pull-out test

**Fig. 3:**
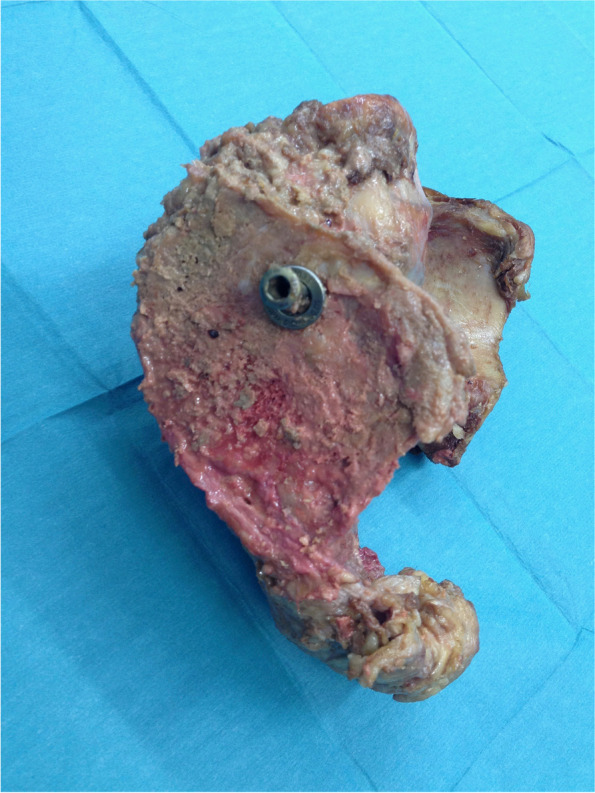
Resection of the ilium before pull-out test to create a plane surface

**Fig. 4:**
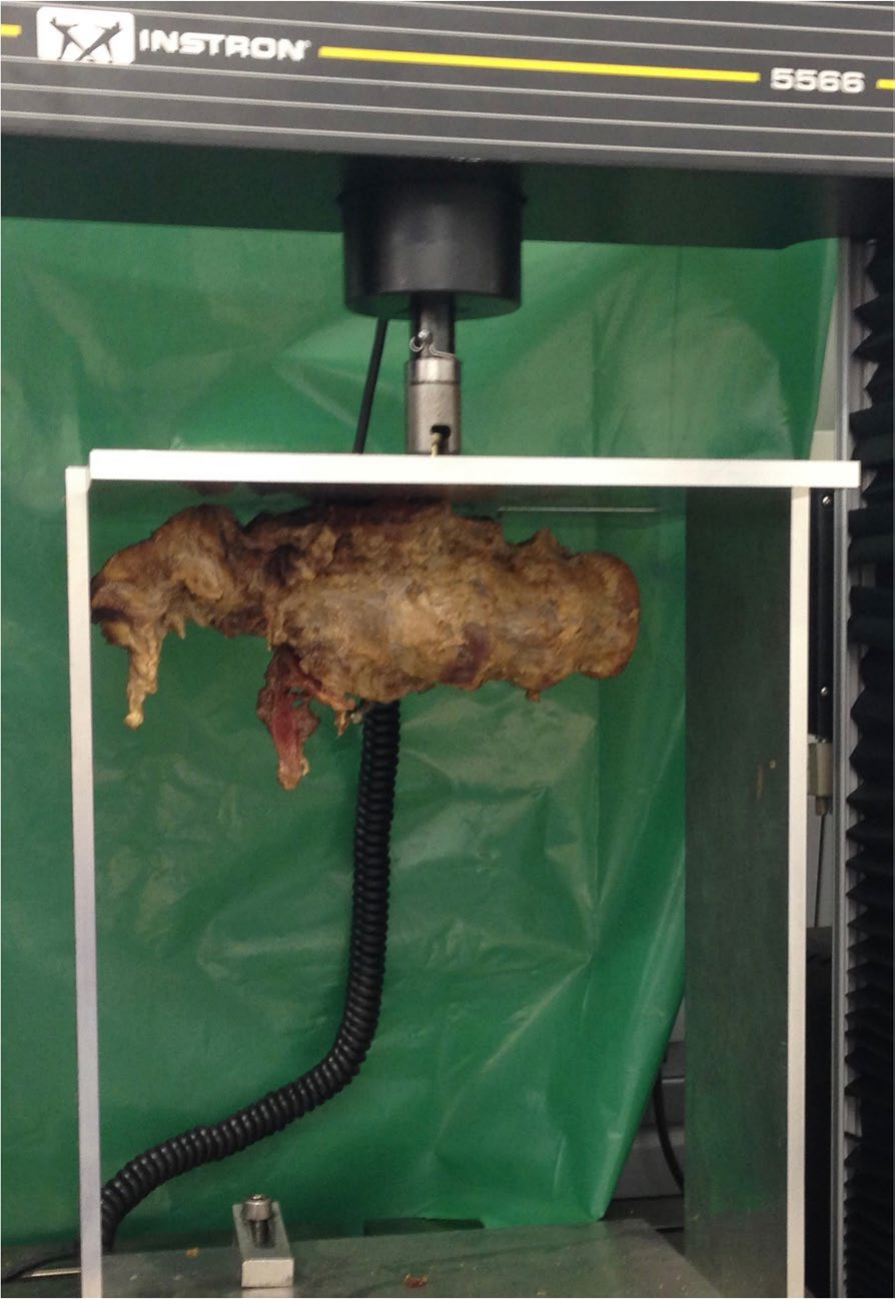
Test setup for the pull-out test

Biomechanical testing was performed after the compression test using an electromechanical material testing machine (Universal Testing Machine, Instron 5566, Instron Corp., Darmstadt, Germany). Before the testing, part of the ilium was resected using the oscillating saw to create a plane surface, above which the screw head protruded 1.5 cm (see Fig. 3). The specimens were then fixated in a custom-made metal holder, and the screw head was inserted in a bracket, which was connected to the testing machine, allowing the screw to be pulled out along its axis of insertion (see Fig. 4). The pull-out test was performed at a rate of 6 mm/min, measuring the maximum pull-out force. The end point was set at 10% loss of strength. The pull-out test was performed starting on the right side in all specimens in order to avoid a negative impact on the results of one group by weakening of the side that was tested second. Consequently, in five of the specimens, the fully threaded screw was tested first, and in the other four specimens the partially threaded screw was tested first.

### Statistical data analysis

Statistical analysis was performed using IBM SPSS Statistics 24 (Statistical Package for the Social Sciences, IBM Corporation, Armonk, NY, USA). Mean values, ranges and standard deviations were calculated for numerous variables. Means were compared using the Wilcoxon test. For all tests, statistical significance was assumed at *p* < 0.05.

### Ethical approval

Institutional review board approval was obtained from the local ethics committee (study number 66/19). All pelvises were acquired from the Anatomic Gift Foundation (Hanover, MD, USA).

## Results

### Compression test

The mean width of the fracture gap was 1.90 mm (SD: 1.64; min: 0.00; max: 4.37) in group A (fully threaded screw) and 0.91 mm (SD: 1.03; min: 0.00; max: 2.66) in group B (partially threaded screw). This difference was statistically significant (*p* = 0.028). Table 1 shows the detailed results of the compression test separated for each specimen.

**Table 1:** Detailed results of the compression test, separated for each specimen (mm)

Specimen	1	2	3	4	5	6	7	8	9
Fully threaded screw	0	2.18	2.67	2.43	0	1.66	0	4.37	3.82
Partially threaded screw	0	0.96	0.87	0	0	1.44	0	2.66	2.27

### Pull-out test

In group A (fully threaded screw), a mean pull-out force of 1696 N (SD: 1452; min: 477; max: 5220) was found; in group B (partially threaded screw), a mean pull-out force of 1616 N (SD: 824; min: 385; max: 3193) was found. This difference was not statistically significant (*p* = 0.767).

Table 2 shows the detailed results of the pull-out test separated for each specimen.

**Table 2:** Detailed results of the pull-out test, separated for each specimen (N)

Specimen	1	2	3	4	5	6	7	8	9
Fully threaded screw	1421	5220	1997	555	2106	1560	624	477	1307
Partially threaded screw	1218	3193	1708	1166	2558	1585	385	1211	1522

## Discussion

The aim of this study was to compare cement-augmented sacroiliac screw osteosynthesis using partially versus fully threaded screws in osteoporotic hemipelvises concerning compression of fracture gap and pull-out force.

Partially threaded cement-augmented sacroiliac screws increased narrowing of the fracture gap significantly compared to augmented fully threaded screws in this biomechanical study in nine osteoporotic hemipelvises with sacral fractures. However, it must be noted that compression of the fracture gap by the partially threaded screws at an average width of 0.91 mm could not be achieved in all pelvises, but only a significant narrowing of the fracture gap, compared to the fully threaded screws. Even if a complete compression of the fracture gap could not be achieved in all pelvises, it can still be assumed that the narrowing of the fracture gap increases stability and thus reduces pain.

Furthermore, there was no significant difference between the pull-out force of augmented partially threaded sacroiliac screws and that of augmented fully threaded screws.

In the current literature, only one biomechanical investigation concerning cement-augmented lag screws in osteoporotic bone can be found. In that study, conducted by Wähnert et al., osteosynthesis with cement-augmented versus non-augmented lag screws was compared in osteoporotic surrogate bones [9]. Therefore, six augmented and six non-augmented screws were compared measuring relaxation after tightening of the screws, relaxation after re-tightening and maximum compression force/maximum torque until failure. The augmented screws showed a significantly less relaxation than the non-augmented screws as well as a significantly less relaxation after loosening and re-tightening. In the stripping test, the augmented screws reached a 94% higher maximum compression and a significantly higher maximum torque [9].

The biomechanical testing in the study by Wähnert et al. differs from our test setup.

However, both studies demonstrate that cement-augmented lag screws can generate interfragmentary compression in osteoporotic bone.

Although there are no studies that compare cement-augmented sacroiliac screw osteosynthesis with partially versus fully threaded screws in osteoporotic bone, some biomechanical studies concerning cement-augmented versus non-augmented sacroiliac screw fixation can be found in the current literature. Suero et al. compared sacroiliac screw fixation with fully threaded screws using a single non-augmented screw, using two non-augmented screws and using a single augmented screw in a biomechanical investigation in 10 human cadaver pelvises, measuring displacement and stiffness of the anterior and posterior part of the pelvis under axial load [3]. Concerning the posterior pelvic ring, no difference was found between the three different techniques, but the single non-augmented screw provided significantly lower stability of the anterior pelvic ring compared to two non-augmented screws and one augmented screw, whereas no significant difference was described between two screws and a single augmented screw [3]. A study by Höch et al. showed no significant difference between sacroiliac screw osteosynthesis with cement augmentation via perforated fully threaded screws and that with non-augmented fully threaded screws, concerning displacement of the fracture and stiffness, in a biomechanical study with eight fresh-frozen pelvises under cyclic loading [10]. Grüneweller et al. found no statistically significant difference in overall construct stability but an increased number of cycles to failure in augmented sacroiliac screw osteosynthesis with fully threaded screws compared with non-augmented fully threaded screws in a hemipelvis model using five fresh-frozen pelvises [8]. A study by Osterhoff et al. with 15 fresh-frozen pelvises showed less screw subsidence and a higher rate of absorbed energy before failure but no difference in screw motion or in the median of cycles until failure for augmented fully threaded sacroiliac screws in S1 and S2, compared with non-augmented fully threaded screws in S1 and S2 and one single transsacral screw [6]. Grechenig et al. found a significantly higher stiffness as well as a significantly higher pull-put force in augmented versus non-augmented partially threaded sacroiliac screws in a biomechanical investigation using six fresh-frozen pelvises [7].

In sum, these studies indicate that cement augmentation improves stability of sacroiliac screw osteosynthesis.

In this context, however, possible cement-associated complications such as cement leakage and cement embolism must also be mentioned [19].

The improvement of stability that can be achieved by the combination of cement augmentation and the compression effect of the lag screw technique seems to make sacroiliac screw osteosynthesis using a single cement-augmented partially threaded screw in S1 sufficient in FFP type-IIB fractures. Using only one instead of two screws eliminates the risk of malpositioning of the second screw, which is more difficult to place than the first one. Furthermore, operative time is shorter when only one screw has to be placed.

### Limitations

Our study is limited by some factors. First, primary stability of the osteosynthesis was measured using a pull-out test. However, pull-out force does not simulate physiological forces applied on a sacroiliac screw during walking. Simulating physiological load application of the pelvis is extremely challenging and thus has to be simplified to some extent for biomechanical testing.

Second, because of the triangular configuration of the generated fracture gap, interfragmentary compression was recorded only by measuring the ventral width of the fracture gap before and after screw osteosynthesis with an electronic measuring gauge. Measurement with a pressure probe would probably have been more accurate, but difficult to implement due to the configuration of the fracture gap.

Third, the mean age of the donors was 68 years and all donors were male. This does not correspond to the typical patient with fragility fracture of the pelvis. However, the specimens were selected because of the limited availability of fresh-frozen pelvises.

In addition, the T-score values of the tested specimens differ from comparative values because bone density was measured at the sacrum and not at the lumbar spine or proximal femur.

Another limitation might be the fact that both sides of the specimens have been used. This procedure has been chosen to allow direct comparison between fully and partially threaded screws in the same specimen, but it might have affected the results of the side that was tested second. To minimize this effect, in five of the specimens the fully threaded screw was tested first, and in the other four specimens the partially threaded screw was tested first.

Finally, the number of specimens used in this study was comparatively small due to the limited availability of fresh-frozen human cadaver pelvises.

## Conclusions

Cement-augmented sacroiliac screw fixation with partially threaded screws resulted in better compression of the fracture gap with similar resistance against pull-out force compared to cement-augmented fully threaded screws.

## Data Availability

All data generated and analyzed during this study are included in this published article.

## Abbreviations

CT: Computed tomography
DXA: Dual-energy X-ray absorptiometry
FFP: Fragility fractures of the pelvis
mL: Milliliter
mm: Millimeter
N: Newton
Nm: Newton meter
SD: Standard deviation
